# Factors influencing the intention of women in rural Ghana to adopt postpartum family planning

**DOI:** 10.1186/1742-4755-10-34

**Published:** 2013-07-22

**Authors:** Sebastian Eliason, Frank Baiden, Gloria Quansah-Asare, Yvonne Graham-Hayfron, Derek Bonsu, James Phillips, Kofi Awusabo-Asare

**Affiliations:** 1 Department of Community Medicine, University of Cape Coast, Cape Coast, Ghana; 2 Centre for Health Research & Implementation Support, Accra, Ghana; 3 Family Health Division, Ghana Health Service, Accra, Ghana; 4 Municipal Director of Health Services, Mfantseman, Ghana; 5 Medical Superintendent, Saltpond Government Hospital, Saltpond, Ghana; 6 Columbia University Mailman School of Public Health, New York, USA; 7 Department of Population and Health, University of Cape Coast, Cape Coast, Ghana

**Keywords:** Postpartum, Family planning, Contraception, Male, Ghana, Sub-Saharan Africa

## Abstract

**Background:**

Uptake of postpartum family planning (PPFP) remains low in sub-Saharan Africa and very little is known about how pregnant women arrive at their decisions to adopt PPFP. This information is needed to guide the development of interventions to promote PPFP.

**Methods:**

We conducted a survey among pregnant women attending antenatal clinics in a rural district in Ghana. We used univariate and multivariate logistic regression analysis to explore how knowledge of various family planning (FP) methods, past experience with their use and the acceptability of PPFP to male partners and close relations influenced the intention of pregnant women to adopt PPFP.

**Results:**

We interviewed 1914 pregnant women in four health facilities. About 84% considered PPFP acceptable, and 70% intended to adopt a method. The most preferred methods were injectables (31.5%), exclusive breastfeeding (16.7%), and oral contraceptive pills (14.8%). Women whose first choice of PPFP method were injectables were more likely to be women who had had past experience with its use (O.R = 2.07, 95% C.I. 1.50-2.87). Acceptability of PPFP by the pregnant woman (O.R. = 3.21, 1.64-6.26), perception of partner acceptability (O.R. = 3.20, 1.94-5.48), having had prior experience with the use of injectables (O.R. = 3.72, 2.61-5.30) were the strongest predictors of the intention to adopt PPFP. Conversely women who knew about the diaphragm (O.R. = 0.59, 0.38-0.93) and those who had past experience with IUD use (O.R. = 0.13, 0.05-0.38) were less likely to want to adopt PPFP.

**Conclusions:**

Acceptability of PPFP to the pregnant woman, male partner approval, and past experience with the use of injectables are important factors in the PPFP decisions of women in this population. Antenatal and early postnatal care need to be adapted to take these factors into consideration.

## Background

The United Nation’s Millennium Development Goal (MDG) 5 aims at reducing maternal mortality by three quarters, between 1990 and 2015. An important intervention towards achieving this target is the promotion of modern family planning (FP) among women in sub-Saharan Africa (SSA) [1,2]. Uptake of modern FP methods remains low in SSA and this is associated with a high incidence of unwanted pregnancies, unsafe abortions, unplanned deliveries and maternal mortalities [1,3].

The periods of pregnancy and immediately after delivery are considered opportune for counseling women on the adoption of modern FP methods. This is because this period is often associated with a woman’s frequent encounter with the health system [4]. These encounters provide avenue to promote optimal spacing of births through postpartum family planning (PPFP) [5]. It has been estimated that PPFP can prevent about 30% and 10% of maternal and child mortalities, respectively [6]. Data from Demographic and Health Surveys (DHS) in 27 countries suggest that less than 35% of women who wish to avoid pregnancy during the postpartum period use any form of modern contraception [7,8]. Very little is known about how pregnant women in SSA arrive at their PPFP decisions. This information is nevertheless critical to the design of strategies to increase the uptake of PPFP [4,9].

### Ghana

In Ghana, unintended childbearing is estimated at about 0.7 births per woman [10]. The major goals of the 1994 Ghana National Population Policy are to reduce the total fertility rate from 5.0 to 3.0, and to increase the contraceptive prevalence rate from 15 to 50 percent between year 2000 and year 2020 respectively [11,12]. According to the 2008 Ghana Demographic and Health Survey [13], fertility rate, contraceptive prevalence rate (CPR) and unmet need in Ghana are 4.0, 17% and 35% respectively, with considerable rural–urban disparities. Women in rural areas have an average of two children more than those in urban areas (4.9 versus 3.1) and use modern FP less than their urban counterparts (15% versus 19%). The level of unmet need for FP methods shows similar trends (rural 38% and urban 32%). The 2011 Multiple Indicator Cluster Survey suggests improvement in the CPR from 17% to 23% and reduction in the level of unmet need from 35% to 26.4%. At current rates, however, the targets set in Ghana’s population policy are unlikely to be met by 2020. The postpartum period remain neglected in FP research in Ghana, and very few studies have focused on the FP needs of women during this period of “window of opportunity” [4,9].

We conducted a survey among pregnant women attending antenatal clinics in rural health facilities in the Central Region of Ghana to identify the factors that influence their intention to adopt PPFP. We report here on how the intention to adopt PPFP is influenced by knowledge of various FP methods, past use of these methods, and the perceived acceptability of PPFP to male partners and close relations.

## Methods

### Study sites

The study was conducted in four health facilities in the Mfantseman District. This district is about 110 km west of Accra and lies along the Atlantic coastline. The district extends 13 kilometers inland, and is essentially rural, except for two towns, Saltpond, and Mankessim, which are semi-rural. The district was selected for this study because of recent reports of increasing incidence of unsafe abortions, high teen pregnancies (13.6% of all pregnancies in 2010) and low uptake of modern FP [14]. It was also based on the familiarity of the research team with the district.

All pregnant women attending antenatal clinic at the Saltpond Government Hospital (semi-rural), Mankessim Health Centre (both semi-rural) and the Biriwa and Anomabo Health Centers (both rural) between January 2012 and April 2012 were targeted and interviewed as they arrived at the facility on routine antenatal visits. The health facilities were purposefully-selected in order to provide a mix of semi-rural and rural settings. We used a five-page questionnaire that explored questions related to the intention to adopt PPFP and the circumstances under which the current pregnancy occurred. Women who indicated that the pregnancy was unexpected, unwanted or both were considered to have had an unplanned pregnancy. Other areas covered in the questionnaire included knowledge and use of various FP methods, their acceptability of PPFP, as well as acceptability to male partners and other close relations (mother, mother-in-law, father-in-law and religious leader). The questionnaire consisted almost entirely of closed-ended questions and was pre-tested in health facilities in the district that were not selected to participate in the study. They were administered by trained research assistants who were familiar with the culture and traditions of the women attending the clinics. The minimum qualification of interviewers was polytechnic or university diploma. The questionnaires were administered in the language that respondents were comfortable to speak in.

### Ethical consideration

Ethical and administrative approvals were obtained from the Ethics Review Committee of the Ghana Health Service (GHS) and the Municipal Health Directorate (MHD). Written informed consent was obtained from each participant.

The data were double-entered using EPI-DATA. The inputs were verified and cleaned to achieve a clean data set. This set was exported into STATA (version 11) for analysis. Descriptive and univariate analyses using logistic regression were performed to explore the influence of knowledge and past use of FP on the intention to adopt PPFP. The effects of perceived PPFP acceptability to male partners and close relations were similarly explored. Factors found in univariate analysis to be significantly associated (P-value <0.05) with the main outcome of interest (intention to adopt PPFP) were included in a multivariate model. Pregnant women were considered to have the intention to adopt PPFP if they indicated they will adopt a modern FP method to delay pregnancy after they deliver.

### Sample size

A total of 1900 pregnant women were targeted to be interviewed on the assumption that 50% of them will have the intention to adopt PPFP. With 80% power, it was possible to estimate the proportion of women willing to adopt PPFP within a margin of error of 3%. The district recorded about 4000 deliveries in 2009.

## Results

### Background of respondents

A total of 1914 pregnant women with an average age of 25.6 years (standard deviation 6.5) were interviewed. The majority were Christians who belonged to the *Fanti* ethnic group. About a fifth had had no formal education and the dominant occupations were petty trading and fish mongering (Table 1).

**Table 1:** Sociodemographic background of respondents

**Variable**		**Intention to adopt PPFP method**	
		Yes (%)*	**No**
Age	≥20 years	1136 (72.6)	429
	*<20 years*	190 (56.7)	145
Respondent-partner age difference	*Partner more than 5 years older*	440 (68.9)	199
	*Partner 1–5 years older*	798 (69.7)	347
	*Partner younger than respondent*	72 (77.4)	21
Highest completed educational level	*Senior high school and above*	127 (56.0)	100
	*Junior high school*	595 (70.9)	244
	*Primary*	317 (74.4)	109
	*None*	286 (70.1)	122
Ethnic group	*Fanti*	1217 (70.4)	513
	*Other*	108 (63.5)	62
Religion	*Christian*	1242 (70.1)	83
	*Other*	529 (29.9)	46
Christian denomination	*Pentecostal and others*	649 (73.3)	236
	*Protestant*	324 (67.9)	153
	*Catholic*	131 (65.4)	68
Occupation	*Fishmonger and petty trader*	1087 (73.2)	399
	*Government and other office workers*	71 (57.7)	52
	*Student*	46 (54.8)	38
	*Other*	68 (65.4)	36
Number of children	*≥Three*	362 (77.9)	103
	*Two*	237 (76.0)	75
	*One*	338 (73.8)	120
	*None*	389 (58.3)	278
Area of residence	*Rural*	652 (71.6)	259
	*Semi-Rural*	673 (68.1)	316
Marital status	*Married*	790 (72.5)	299
	*Engaged or cohabiting*	380 (69.5)	167
	*Single, divorced, separated or widowed*	152 (58.7)	107

*Row percentages.

About a third (35.2%) of pregnant women did not have a child. Of those who had at least one child, the average number of children was 2 (standard deviation-2). The majority of women (70%) indicated that the pregnancy they were carrying was unexpected or unwanted or both at the time it occurred. About 70% of pregnant women expressed the intention to adopt PPFP. For women who preferred to have more children, the average desired time before a next pregnancy was 4.6 years (standard deviation 1.5 years). The most preferred PPFP methods were injectables (31.5%), exclusive breastfeeding (16.7%), and oral contraceptive pills (14.8%). Women whose first choice of PPFP method were injectables were more likely to be women who had had past experience with its use (O.R = 2.07, 95% C.I. 1.50-2.87). Past use was however less predictive in the case of the pill (1.19, 0.81-1.75). It actually appeared to be inhibitory in the case of exclusive breastfeeding (0.81, 0.42-1.57).

### Effect of knowledge of various FP methods

The two most widely known FP methods were the male condom (86.4%) and injectables (80.5%) while the least known were the foaming tablets (20.7%) and the diaphragm (24.4%). Respondents who had heard about the pill (1.76, 1.40-2.20), injectables (2.20, 1.73-2.79), implants (1.60, 1.30-1.95), male (1.97, 1.50-2.59) and female condom (1.62, 1.29-2.03) were more likely to want to adopt PPFP. Participants who were aware that exclusive breastfeeding (1.83, 1.34-2.50) could be a method of contraception were also more likely to want to adopt PPFP. On the hand, those who had heard of the diaphragm (0.74, 0.59-0.92) and foaming tablets (0.77, 0.61-0.98) were unlikely to have the intention of adopting PPFP. In multivariate analysis, knowledge of the use of implants (O.R. = 1.64, 0.11-2.41) and exclusive breastfeeding (O.R. = 1.56, 1.12-2.18) were associated with a intention to adopt PPFP while knowledge of the diaphragm (O.R. = 0.59, 0.38-0.93) was associated with the lack of intention to adopt PPFP (Table 2).

**Table 2:** Effect of knowledge of family planning on intention to adopt PPFP

**Method**		**Intention to adopt PPFP method**		**Univariate**	**Multivariate**
				**O.R. (95% C.I.)†**	**O.R. (95% C.I.)†**
		**Yes**	**No**		
**Oral contraceptive pills**	Yes	1069	405	1.76 (1.40-2.20)	1.16 (0.70-1.91)
	No	257	171	1.00	1.00
**Intrauterine contraceptive device**	Yes	490	232	0.87 (0.71-1.06)	N/A
	No	836	344	1.00	
**Injectables**	Yes	1124	413	2.20 (1.73-2.79)	1.37 (0.79-2.41)
	No	202	163	1.00	1.00
**Implants**	Yes	881	319	1.60 (1.30-1.95)	1.64 (1.11-2.42)
	No	445	257	1.00	1.00
**Male condom**	Yes	1184	466	1.97 (1.50-2.59)	1.18 (0.58-2.41)
	No	142	110	1.00	1.00
**Female condom**	Yes	1068	414	1.62 (1.29-2.03)	1.03 (0.60-1.75)
	No	258	162	1.00	1.00
**Diaphragm**	Yes	300	164	0.74 (0.59-0.92)	0.59 (0.38-0.93)
	No	1025	412	1.00	1.00
**Foaming tablets**	Yes	258	137	0.77 (0.61-0.98)	0.84 (0.53-1.32)
	No	1067	258	1.00	1.00
**Rhythm (calendar) method**	Yes	757	356	0.82 (0.67-1.01)	N/A
	No	568	220	1.00	
**Withdrawal method**	Yes	854	345	1.21 (0.99-1.48)	N/A
	No	471	231	1.00	
**Emergency contraception**	Yes	630	262	1.09 (0.89-1.32)	N/A
	No	695	314	1.00	
**Exclusive breastfeeding**	Yes	567	205	1.83 (1.34-2.50)	1.56 (1.12-2.18)
	No	136	90	1.00	1.00

### Past use of various methods

The most widely used FP methods were the withdrawal (29.5%) and male condom (26.9%) while the least used were the diaphragm (1.0%) and foaming tablets (1.2%). Respondents who had used the pill (O.R = 2.53, 95% C.I. 1.83-3.49), injectables (3.83, 2.70-5.43) and emergency contraceptive pills (1.52, 1.05-2.20) in the past were more likely to want to adopt PPFP. On the hand, those with prior experience with the use of the IUD (0.39, 0.16-0.96) were unlikely to want to adopt PPFP. In multivariate analysis, women who had used injectables (O.R. = 3.72, 2.61-5.30) and pill (O.R. = 2.22, 1.59-3.11) were more likely to have the intention to adopt PPFP while those who had prior experience with IUD use (O.R. = 0.13, 0.05-0.38) were significantly less likely to have the intention of adopting PPFP (Table 3).

**Table 3:** Effect previous use of family planning on intention to adopt PPFP

**Method**		**Intention to adopt PPFP method**		**Univariate**	**Multivariate**
				**O.R. (95% C.I.)†**	**O.R. (95% C.I.)†**
		**Yes**	**No**		
**Oral contraceptive pills**	Yes	257	<0.01	2.53 (1.83-3.49)	2.22 (1.59-3.11)
	No	1069		1.0	1.00
**Intrauterine contraceptive device**	Yes	9	0.03	0.39 (0.16-0.96)	0.13 (0.05-0.38)
	No	1317		1.0	1.00
**Injectables**	Yes	301	<0.01	3.83 (2.70-5.43)	3.72 (2.61-5.30)
	No	1025		1.0	1.00
**Implants**	Yes	19	0.62	0.82 (0.38-1.78)	NA
	No	1307		1.0	
**Male condom**	Yes	358	0.97	1.00 (0.81-1.25)	NA
	No	968		1.00	
**Female condom**	Yes	22	0.37	0.73 (0.37-1.46)	NA
	No	1304		1.00	
**Diaphragm**	Yes	12	0.53	0.74 (0.29-1.71)	NA
	No	1313		1.00	
**Foaming tablets**	Yes	13	0.43	0.70 (0.29-1.71)	NA
	No	1312		1.00	
**Rhythm (calendar) method**	Yes	306	0.25	0.87 (0.70-1.10)	N/A
	No	1019		1.0	
**Withdrawal method**	Yes	407	0.09	1.20 (0.97-1.50)	N/A
	No	918		1.0	
**Emergency contraception**	Yes	135	0.02	1.52 (1.05-2.20)	1.26 (0.86-1.85)
	No	1190		1.0	1.00
**Exclusive breastfeeding**	Yes	19	0.08	1.54 (0.27-1.08)	NA
	No	1307	561	1.00	

### Acceptability of PPFP to partners and close relations

The majority (84%) of pregnant women considered it acceptable for women (self-approval) to use modern FP methods in PPFP and 70.0% had the intention to adopt a method. Although self-approval was strongly associated (8.55, 6.29-11.63) with the intention to adopt a method, 64% of women who considered the PPFP acceptable did not have the intention to adopt it. Further analysis show the more educated a pregnant woman was, the more likely she was to consider PPFP acceptability and yet not intend to adopt it (P-value for trend <0.01). Pregnant women with higher education (beyond secondary school) were nearly four times (3.87, 2.57- 5.84) as likely to make this consideration as women who had had no formal education. This seemingly discordant consideration was neither associated with the age of a pregnant woman nor with the number of children she had.

The majority (76.2%) of pregnant women perceived that their partners will consider their adoption of PPFP acceptable. A higher proportion (82.0%) indicated that they will require the permission of their partners before they actually adopted a method. Among women who perceived that PPFP will be acceptable to the partners, 82.3% thought they will still need their permission before they could adopt a method. Also most (90.5%) women who indicated they personally approved of PPFP still considered that they will need the approval of their partners before they could adopt it. In univariate analysis, the intention to adopt PPFP was strongly associated with the perceived acceptability of PPFP by partners and close relations (P-value < 0.01 in all cases). After adjustments in multivariate analysis however, only self-approval of PPFP (3.21, 1.64-6.26) and perceived acceptability by male partner (3.2, 1.94-5.48) were significantly associated with the intention to adopt PPFP (Table 4).

**Table 4:** Effect of acceptability of PPFP and related factors on intention to adopt PPFP

			**Intention to adopt PPFP method**		**Univariate**	**Multivariate**
			**Yes**	**No**	**O.R. (95% C.I.)†**	**O.R. (95% C.I.)†**
Acceptability of PPFP to respondent and relations	Pregnant woman	Yes	1244	372	8.55 (6.29-11.63)	3.21 (1.64-6.26)
		No	79	202	1.00	
	Partner	Yes	950	193	7.78 (5.81-10.40)	3.2 (1.94-5.48)
		No	138	218	1.00	
	Mother	Yes	625	175	3.30 (2.50-4.33)	1.60 (0.78-3.27)
		No	192	177	1.00	
	Mother-in-law	Yes	423	110	3.89 (2.84-5.33)	3.06 (0.71-13.27)
		No	168	170	1.00	
	Father-in-law	Yes	377	99	4.19 (3.01-5.83)	0.56 (0.12-2.65)
		No	151	166	1.00	
	Religious leader	Yes	473	154	2.75 (2.07-3.63)	0.90 (0.43-1.87)
		No	198	177	1.00	
Religious partner’s permission before adopting PPFP		Yes	1136	187	2.23 (1.75-2.85)	0.94 (0.54-1.61)
		No	419	154	1.00	
Willing to use postpartum without making partner aware		Yes	614	127	3.04 (2.41-3.83)	1.37 (0.90-2.11)
		No	709	446	1.00	

## Discussion

Although fewer than 30% of women in this study had ever used a modern FP method, 84% considered PPFP acceptable and 70% expressed the intention to adopt a method. This seemingly favorable basis for the promotion of PFPP needs however to be appreciated with caution. This is because of the existence of the well-documented wide gap between FP intentions and actual uptake among women in SSA [8,15]. In this study, findings have been made that suggests that what is likely to happen among the cohort of women interviewed would not be substantially different from what has been found among non-pregnant women interviewed in the GHDS and others studies in SSA; namely a low level of uptake despite a high level of expressed intention to use. The wide disparity between pregnant women’s level of knowledge of various modern FP methods, and their use of these methods in the past (Tables 2 &3) is evidence in this regard. Furthermore the fact that even among the 85.2% of women who considered PPFP to be acceptable practice, 64% did not have the intention to adopt a method is pointer to the fact that the declared intention to adopt PPFP is predicated on a number of conflicting factors. An important limitation of this study therefore is the lack of follow-up of the pregnant women to establish how their PPFP intentions were met, and to compare the characteristics of those who take up PPFP methods and those who do not.

### Personal conviction versus role of male partners

The study identified personal conviction as important in getting pregnant women to have the intention to adopt PPFP. This finding makes a case for continuing the education of pregnant women on the benefits of PPFP, and the advantages of spacing deliveries. Further evidence from the study however suggests that personal conviction is insufficient to ensure actual uptake of PPFP by the interviewed women. This is because, by even greater measure, the women in this study made the point about their need for partner approval before they could adopt a method of PPFP. Similar studies in SSA have pointed to the critical role played by male partners in the FP decision-making processes of women [16-18]. The finding in this study is further indication of how deep-seated the influence of male partners is on the decision of women in SSA to adopt FP. The evidence suggests that the personal conviction of women is likely to be superseded by the influence of partner approval.

What is however an irony is that in various surveys among men in SSA, it has been shown that except in except in polygamous societies, male partner acceptability of FP is not particularly different from that of women [19]. It has been shown that men are as desirous as their female partners to limit the number of children they father. It would appear therefore that women have an inaccurate perception of their male partners about FP. This has been hypothesized to be the result of inadequate spousal communication about FP [20]. Although the findings of the analysis of DHS data in Ghana and Chad [15,21] cast doubt on the likely effectiveness of spousal communication in causing an increase in the uptake of contraceptive methods, other studies [18], including recent intervention studies have provided convincing evidence about the effectiveness of using spousal communication to cause an increase in the uptake of FP. A trial in Malawi demonstrated that spousal communication on FP led to men actually facilitating contraceptive use by their partner [22]. Spousal communication is now considered to be an integral component of successful interventions to increase male involvement in FP in SSA [23]. The challenge that remains however is how to reach out to male partners and facilitate informed FP spousal discussions. Studies in Uganda and South Africa have shown that a simple intervention such as written letter of invitation to a male partner can lead to significant increase in male attendance at antenatal clinics and opportunities for couple counseling [24,25]. Consistent with the need to have a more aggressive approach to ensuring active male participation in PPFP, there is the urgent need to identify additional approaches that will contribute to assuring women of spousal support of their intentions to adopt PPFP.

### Past use of modern family planning

In this study, we found that the intention to adopt PPFP and the selection of injectables as the method of choice were both significantly influenced by past use of the method. The apparent appreciation of injectables is corroborated by the findings of the 2008 Ghana DHS and a recent review of the uptake of various FP methods in SSA [13,26]. An investigation of the factors that make women in Ghana prefer injectables would most likely provide indications of the interventions that would be required to increase the uptake of PPFP in general. The possibility that injectables make it possible for women to use FP discretely needs to be considered.

In the 2008 Ghana DHS, the proportion of married non-users of FP who indicated the intention to use FP ranged from 43.7% to 53.0% [13]. This range is much lower than the 70% found in this study and this could be due to the fact that all the respondents in this study were pregnant women who would reasonably be expected to have a greater desire to delay repeat pregnancy. Notwithstanding the limitation of using intentions to gauge likely uptake, this finding support the need for pregnant women to be particularly targeted in the promotion of FP in this population.

### Implications for the health system in Ghana

The model for delivering antenatal (including community-based) services in Ghana has seen very little innovation and responsive to the evidence on the need for male participation. The focus has remained on women despite clear evidence that the continued limited impact of the family planning program in Ghana is due largely to the continued neglect of men as equal target [27]. Using a cross-sectional design this study has demonstrated that male partner approval is central to getting pregnant women to adopt PPFP. To facilitate this, a mandatory session of couple counseling should be actively explored by health workers as part of the routine antenatal care of each pregnant woman. It should be made a part of standard ANC protocol and health workers required to ensure adherence during facility and community-based care.

Making the correct choice of PPFP method and receiving the appropriate counseling on it is critical to retaining confidence in the use of the injectables in PPFP. The use of injectables in Ghana is associated a number of health-related myths and misconceptions that need to be addressed [28]. A common misconception is that injectables reduce breast milk and should not be used postpartum until menstruation resumes. To the extent that the injectable is emerging as a most highly-favored method of FP, including PPFP, there is the need for injectable-specific, targeted training for health workers involved in PPFP counseling. Non-pregnant women who opt to either start or discontinue injectables in routine family planning clinics should be explicitly informed that the method can be safely used in PPFP.

## Conclusion

Male partner approval, past experience with the use of injectables and personal approval of PPFP are the major determinants of the intention of pregnant women in this population to adopt PPFP. Procedures adopted at antenatal and postnatal clinics should be adapted to take these factors into consideration.

## Competing interests

The authors declare no conflict of interest.

## Authors’ contributions

FB was responsible for the conceptualization of the study. SE, FB, YGH, DB were responsible for its design, implementation and production of the initial draft of the manuscript. SE and FB were responsible for finalizing the manuscript after critical review by GQA, JP and KAA. All authors read and approved the final manuscript.
